# Photodynamic therapy in oral lichen planus: A prospective case-controlled pilot study

**DOI:** 10.1038/s41598-020-58548-9

**Published:** 2020-02-03

**Authors:** Raluca Cosgarea, Robert Pollmann, Jusra Sharif, Thomas Schmidt, Ronja Stein, Aura Bodea, Thorsten Auschill, Anton Sculean, Rüdiger Eming, Brandon Greene, Michael Hertl, Nicole Arweiler

**Affiliations:** 1 Department of Periodontology, Philipps-University Marburg, Marburg, Germany; 2 Clinic for Prosthodontics, University of Medicine and Pharmacy, Faculty of Dentistry “Iuliu Hatieganu”, Cluj-Napoca, Romania; 3 Department of Dermatology und Allergology, Philipps-University Marburg, Marburg, Germany; 4 Periodontal Private Practice, Cluj-Napoca, Romania; 5 Department of Periodontology, University of Bern, Dental School, Bern, Switzerland; 6 Institute of Biometry and Statistics, Philipps-University Marburg, Marburg, Germany

**Keywords:** Gum disease, Clinical trials

## Abstract

Oral lichen planus (OLP) is a common, chronic relapsing inflammatory disorder of the mucous membranes, which causes major discomfort. Current treatment includes topical/systemic glucocorticoids, immune modulators and systemic immunosuppressants, which may lead to considerable side-effects. The aim of this study was to determine the clinical and immunological efficacy of photodynamic therapy (PDT) in OLP as an alternative, easy-to-use, safe and non-invasive treatment. Twenty patients with OLP were treated with PDT in a prospective case-controlled pilot-study. PDT was performed on the most extensive oral lesion in 4 sessions (day 1, 3, 7, 14). Peripheral blood and lesional T cells were analysed before (day 1) and after PDT treatment (day 28). PDT led to a statistically significant reduction of clinical parameters (lesion size, ABSIS, Thongprasom-score) and improvement of all evaluated quality-of-life (QOL) items. The clinical improvement was accompanied by a significant decrease of the relative number of CD4+ and CD8+ T cells in mucosal OLP-lesions. Furthermore, CXCL10 plasma levels were decreased and the number of activated peripheral CD4 + CD137+ and CD8 + CD137+ T cells and IL-17-secreting T cells was diminished. PDT treatment in OLP leads to lesion reduction and improvement of QOL, and induces local and systemic anti-inflammatory effects. The study identifies PDT as a novel therapeutic option in OLP.

## Introduction

Lichen planus (LP) is a common T cell mediated, chronic inflammatory disorder which affects the oral mucosa and causes major discomfort, its etiology is still not entirely elucidated^1–4^. Oral LP (OLP) affects the buccal mucosa, tongue and gingiva, and usually presents with symmetrical, bilateral or multiple lesions^5,6^. The erosive and atrophic forms may present with remarkable burning and pain sensations that restrict food intake and performance of oral hygiene measures leading to weight loss, nutritional deficiencies^7,8^, loss of teeth and function with a major impact on the quality of life (QOL). These OLP forms are often resistant to treatment^9,10^ and have a higher tendency to malignant transformation^11,12^.

At present, topical corticosteroids are widely accepted as the primary choice of OLP therapy, but other therapeutical protocols have also been investigated, i.e. topical and systemic retinoids, calcineurin inhibitors, aloe-vera, and thalidomide^6,7,13–25^. However, some of these treatment options proved to be ineffective or only partially effective^12^. Photodynamic therapy (PDT) as an easy-to-use and safe treatment option induces cell and tissue damage by combining the use of a photosensitizer and light that activates the photosensitizer by exposure to low-level visible light in an appropriate wavelength. The general principle of PDT was first described in 1900 by the work of Raab and Tappeiner who evaluated the effects of acridine on malaria-causing protozoa showing a lethal effect of this combination on a species of paramecium (Infusoria)^26^. They discovered the optical property of fluorescence and that one of its products was responsible for the cytotoxic effects *in vitro* which relies on the energy transfer from light to the chemical, similar to the plants (light-chlorophyll absorbtion). This effect was later applied by Tappeiner and Jesionek for the treatment of skin cancers and described the therapy as “photodynamic phenomenon”^27^. For the successful inactivation of bacteria, Jodlbauer and Tappeiner, and Huber demonstrated that oxygen was a prerequisite for the photosensitization reactions^28,29^. Based on these early studies PDT was then used for the treatment of actinic keratosis and various types of skin cancers (such as basal cell carcinoma) and in the past 15 years for the treatment of OLP.

The human tissue transmits efficiently red light and this combined with a longer activation wavelength of a photosensitizer leads to a deeper light penetration^30^. Most photosensitizers allow a light penetration of 0.5 cm (for 630 nm) to 1.5 cm (for ab. 700 nm)^31,32^. According to these properties, the therapeutical effect of various photosensitizers is defined for different pathological conditions and tumors. Thus, for each tissue and photosensitizer a different total light dose, dose rate and tissue destruction is achieved^30^. Light radiation in a specific wavelength for the photosensitizer, sends the photosensitizer from a low-energy ground state to an excited singlet state. This may afterwards undergo a transition to a higher energy triplet state which reacts with the endogenous oxygen. Thus, singlet oxygens and cytotoxic free radicals are released and determine membrane lysis, destruction of targeted cells, and inactivation of proteins^13,22^. The cytotoxic effects of PDT rely on the fact that during light radiation, photosensitizers that localize in lysosomes and cell membranes induce necrosis, while those penetrating mitochondria lead to apoptosis^33^. Additionally, PDT seems to induce complex inflammatory and immune responses^34^. In murine tumor models a strong invasion of neutrophils, mast cells and monocytes had been observed during and after PDT^35^ accompanied by activation of specific T-lymphocytes^36^ and apoptosis in the hyperproliferating inflammatory cells^37,38^.

PDT has been increasingly used for treating various types of oral cancer, mucosal hypertrophy, leukoplakia or erythroplakia showing no to optimal efficacy (no response to treatment to complete remission)^39–43^. A few studies investigated the efficacy of PDT in reducing the clinical symptoms of OLP, i.e. lesion size and symptoms, and found mixed clinical responses^22,44–49^. Furthermore, limited evidence exists regarding the histological, immunological effects of PDT in OLP. The aim of this study was to determine the efficacy of PDT in OLP based on the combination of clinical and immunological parameters. The results show a reduction of lesion size and decrease of ABSIS and Thongprasom scores and an improvement of QOL parameters. These effects are accompanied by a decrease of CD4^+^, CD8^+^ and IL-17^+^ cells in OLP lesions. In peripheral blood, PDT induces a decrease of CD4^+^CD137^+^ and CD8^+^CD137^+^ and IL-17^+^ T cells and CXCL10 plasma levels.

## Results

### PDT treatment is accompanied by clinical amelioration of OLP

**Figure 1:**
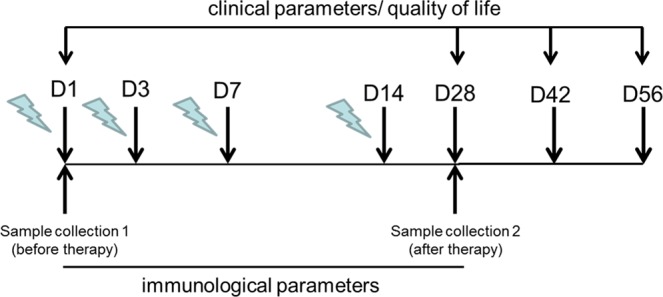
Time line of PDT treatment. Clinical and QOL parameters were assessed on day 1 (D1; before therapy), day 28 (D28, 14 days after therapy), day 42 (D42, 28 days after therapy) and day 56 (D56; 42 days after therapy). Samples were collected at D1 and D28. PDT, photodynamic treatment; QOL, quality of life.

**Figure 2:**
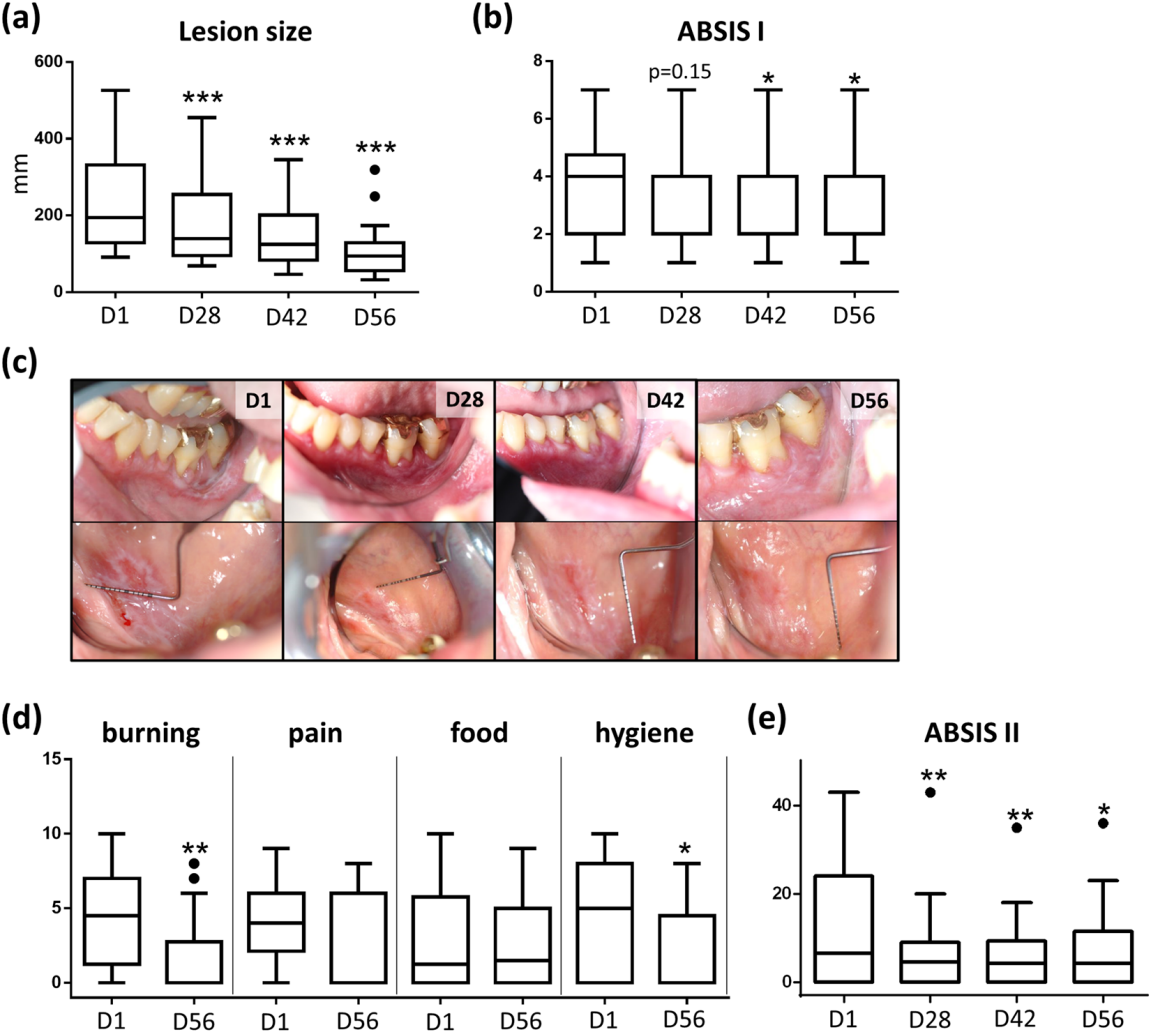
PDT leads to clinical amelioration of OLP. (**a**) PDT induces a reduction of lesion size over time (D1-D56). (**b**) PDT leads to reduction of ABSIS score I over time (D1-D56). (**c**) Photographically documentation of lesion size reduction over time (D1-D56). (**d**) PDT leads to a reduction of ABSIS II score over time (D1-D56). (**e**) QoL parameters (burning, pain, food, oral hygiene) are improved after PDT treatment (D1-D56). *p < 0.05, **p < 0.01, ***p < 0.001. PDT, photodynamic treatment; QOL, quality of life.

Mucosal lesions of 20 patients with OLP were treated with PDT within 14 days (4 sessions; day 1, 3, 7, and 14; Fig. 1) and clinical parameters were assessed at day 1 (baseline) and day 28, 42, and 56. PDT treatment led to a highly significant size (*P* < 0.001) reduction of the mucosal lesion starting from 14 days after PDT (day 28; Fig. 2a,c) that correlated with a significant improvement of the ABSIS I score (*P* < 0.05) at days 42 and 56 (Fig. 2b). Stratification of patients with moderate (≤5 years; n = 9) and long-lasting disease duration (>5 years; n = 11, see Table 1) showed a similar reduction of the lesion size and ABSIS I scores though statistical differences could not be detected due to the reduced patient numbers in the groups (see Supplementary Fig. S1). Six weeks after PDT (day 56), two scores reflecting treatment efficacy^50,51^ showed a shift from more severe lesions (Thongprasom score 3,4,5) to lesions with softer forms (score 1,2,3) or to complete/partial remission (Carrozzo-Gandolfo score) in the majority of patients (Table 2). At day 56, all evaluated items for QOL showed improvements. Statistically significant however, was the decrease of the burning sensation and self-performed oral hygiene (Fig. 2d, *P* = 0.003) in concordance with decreasing ABSIS II scores at follow-ups compared to baseline (Fig. 2e, *P* = 0.005) which was most prominent in patients with long-lasting disease duration (see Supplementary Fig. S1).

**Table 1:** Clinical description of the studied patient cohort with OLP.

**No. of subjects (n)**	20
**Gender male/female (n)**	3/17
**Age (years)**	62 ± 8.66
**Disease duration (no. patients)**	
<1 year	1
1–5 years	8
5–10 years	2
>10 years	9
**Location of treated OLP lesion**	
Lining of mucosa cheek	6
Gingiva	14

**Table 2:** Evaluation of treatment response according to Thongprasom, and Carrozzo and Gandolfo scores^50,51^.

Variables	day 1	day 28	day 42	day 56
**Thongprasom score N (%)**				
Score 0	0 (0%)	0 (0%)	0 (0%)	0 (0%)
Score 1	0 (0%)	1 (5%)	4 (20%)	6 (30%)
Score 2	0 (0%)	8 (40%)	9 (45%)	11 (55%)
Score 3	5 (25%)	9 (45%)	7 (35%)	3 (15%)
Score 4	11 (55%)	2 (10%)	0 (0%)	0 (0%)
Score 5	4 (20%)	0 (0%)	0 (0%)	0 (0%)
**Carrozzo-Gadolfo score N (%)**				
Complete remission	—	2 (10%)	5 (25%)	7 (35%)
Partial remission	—	14 (70%)	13 (65%)	12 (60%)
No response	—	4 (20%)	2 (10%)	1 (5%)

### PDT determines an insignificant reduction of the oral bacteria

Two weeks after the final PDT session (day 28), There was a quantitative reduction of the majority of the investigated oral bacteria. Nevertheless, none was significantly reduced (Table 3).

**Table 3:** Comparison of the microbial load (oral bacteria) before and after PDT.

Oral bacteria	p-value between timepointsT1-T4
*A. actinomycetemcomitans*	1.000
*A. viscosus*	1.000
*T. forsythia*	0.689
*C. rectus*	0.500
*T. denticola*	0.506
*E. corrodens*	0.219
*P. intermedia*	0.376
*P. micra*	0.126
*P. gingivalis*	0.627
*F. nucleatum*	1.000
*A. odontolyticus*	0.501
*Capnocytophaga sp.*	1.000
*C. concisus*	0.727
*E. nodatum*	1.000
*S constellatus group*	0.109
*C. gracilis*	0.500
*S. mitis group*	0.252
*P. nigrescens*	0.291
*S. gordonii group*	0.508
*V. parvula*	1.000

### PDT leads to a decrease of peripheral CD4^+^CD137^+^ and CD8^+^CD137^+^ and IL-17^+^ T cells and CXCL10 plasma levels

**Figure 3:**
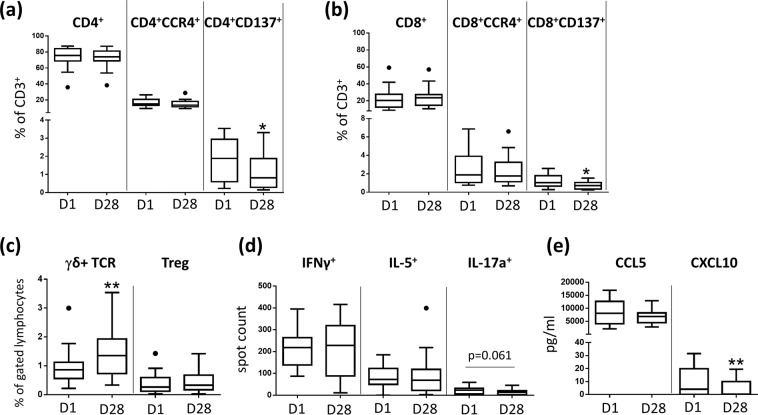
PDT induces systemic anti-inflammatory effects in OLP. (**a**) Relative number of CD137 expressing peripheral blood CD4+ T- cells is decreased 14 days after PDT treatment. Results based on analysis by flow cytometry. (**b**) Relative number of CD137 expressing peripheral blood CD8+ T- cells is decreased 14 days after PDT treatment. Results based on analysis by flow cytometry. (**c**) PDT leads to an increase of the relative number of peripheral blood γδ T cells. Results based on analysis by flow cytometry. (**d**) Number of peripheral blood IL-17a producing T cells is decreased 14 days after PDT treatment. Results based on ELISpot. analysis. **(e)** CXCL10 plasma levels are decreased 14 days after PDT treatment. Results based on ELISA analysis.*p < 0.05, **p < 0.01. PDT, photodynamic treatment.

**Figure 4:**
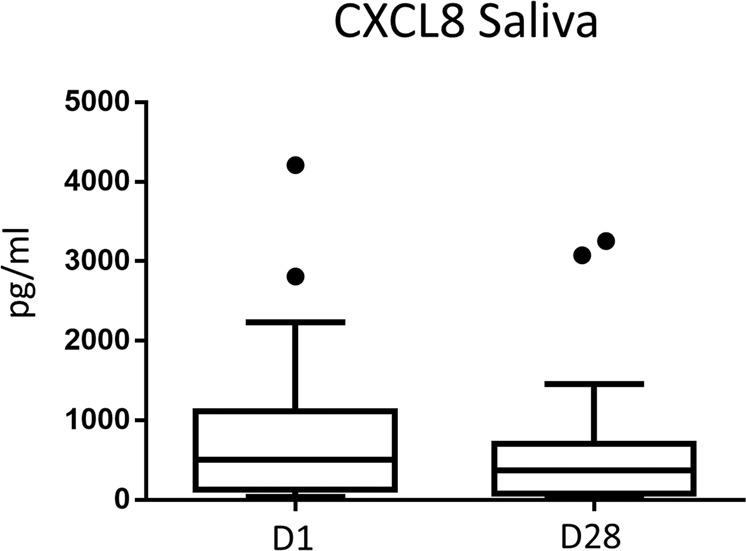
CXCL8 saliva levels are slightly decreased after PDT treatment. Shown are saliva concentrations (pg/ml) of CXCL8 before (day 1, D1) and after (day 28, D28) PDT treatment of oral OLP lesions.

Fourteen days after the last PDT treatment (day 28), relative numbers of peripheral blood CD4^+^ T cells or CD8^+^ T- cells remained stable (Fig. 3a,b). Additionally, the relative numbers of CD4^+^ and CD8^+^ T- cells expressing the skin-homing factor CCR4 were not altered. In contrast, after PDT treatment, the relative numbers of CD137^+^ (activated) CD4^+^ and CD8^+^ cells were significantly decreased (Fig. 3a,b). Furthermore, the relative number of peripheral γδ^+^ T- cells was significantly increased whereas the relative number of CD4^+^CD25^+^CD127^low^ T regulatory cells remained unaffected (Fig. 3c). ELISpot analysis revealed a consistent number of peripheral blood Th1 (IFNγ^+^) and Th2 (IL-5^+^) cells after PDT while peripheral Th17 (IL-17a^+^) cells declined (*P* = 0.061; Fig. 3d). CCL5 plasma levels were unaffected whereas CXCL10 plasma concentrations were significantly decreased (Fig. 3e). CXCL8 plasma levels were below the detection limit (data not shown). In contrast, in saliva of treated OLP patients, CXCL8 concentrations were slightly but not significantly lowered (Fig. 4). CCL5 and CXCL10 saliva concentrations were below the detection limit (data not shown).

### PDT leads to a reduction of infiltrating CD4^+^ and CD8^+^ T- cells in mucosal lesions

**Figure 5:**
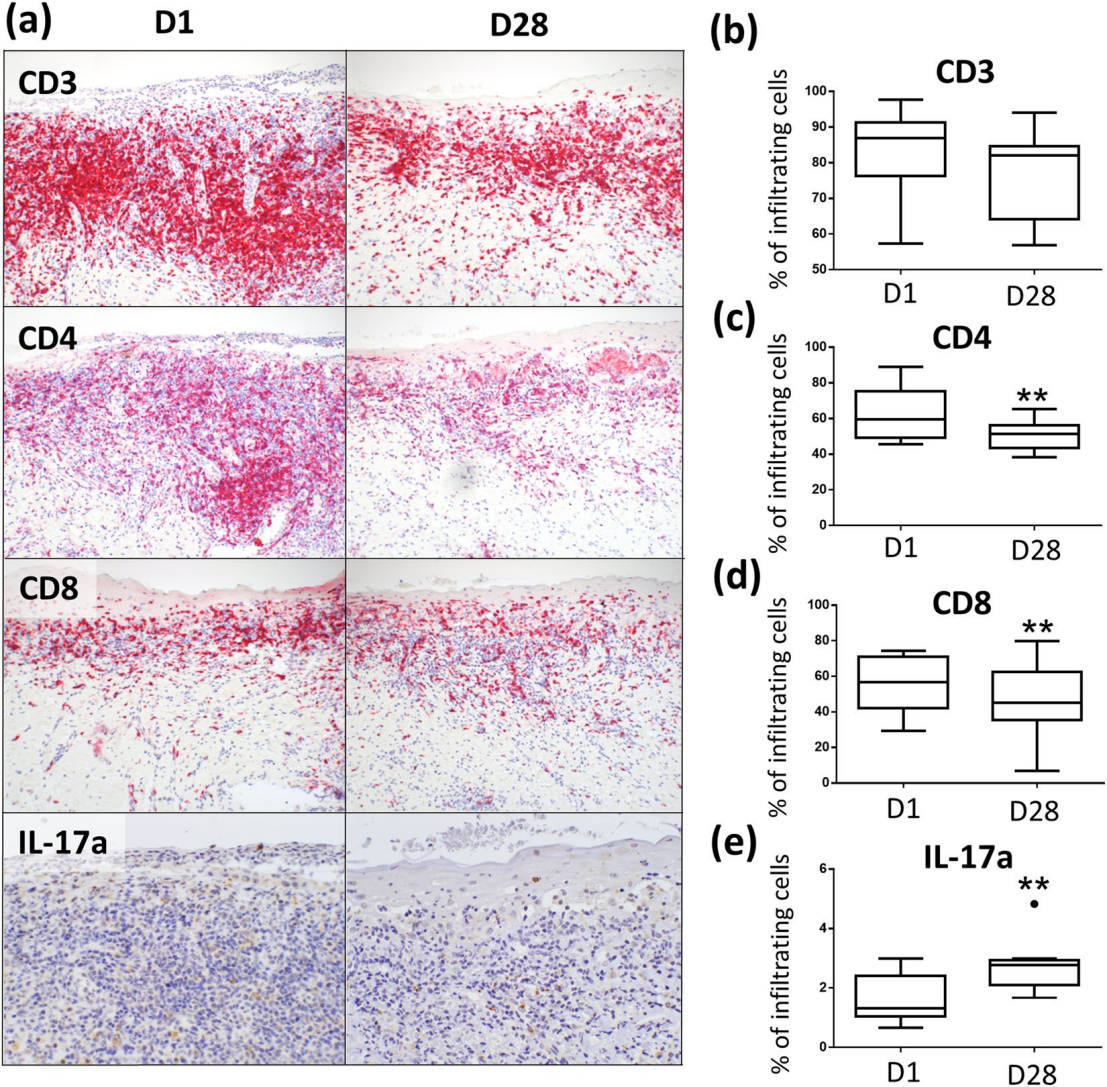
PDT induces local anti-inflammatory effects in OLP. (**a**) Overview of immunohistochemical staining of mucosal tissue sections with a lesional T- cell infiltrate before and after PDT treatment (D1, D28). (**b–d**) Relative number of lesional CD3+, CD4+ and CD8+ cells are decreased 14 days after PDT treatment. (**e**) Relative number of lesional IL-17+ cells is increased 14 days after PDT treatment. **p < 0.01. PDT, photodynamic treatment.

Immunohistologically, mucosal lesions of OLP showed the characteristic T cell-dominated subepidermal band-like infiltrate (Fig. 5a). Lesional CD4^+^ T- cells were widely spread in the upper dermis, particularly at the perivascular region, while lesional CD8^+^ T -cells were mainly located in a band-like pattern along the basal membrane zone (BMZ). IL-17a^+^ T -cells were also found along the BMZ and at the perivascular regions (Fig. 5a) as recently described^52^. After PDT, the CD3^+^ lesional T- cell infiltrate was markedly but not significantly diminished at day 28, while the relative numbers of skin infiltrating CD4^+^ T cells and CD8^+^ T- cells were significantly decreased (Fig. 5b–d). Of note, PDT led to an altered redistribution of dermal CD8^+^ T- cells from the BMZ to the perivascular area (Fig. 5d). Notably, the relative number of IL-17a^+^ cells was significantly increased in mucosal lesions after PDT treatment (Fig. 5e).

## Discussion

PDT treatment led to a highly significant reduction of the size of OLP lesions, also reflected by a significant decrease of the ABSIS scores. Moreover, treatment efficacy was evident by the increasing numbers of mucosal lesions showing complete remission (day 28: 10%; day 56: 35%), also reflected by decreasing Thongprasom scores at day 56 compared to baseline (Table 2). These results are in concordance with reports by others who used PDT in treating OLP and showed marked improvement of the lesion size^22,45–47,49,53^. Furthermore, the results of our study show a marked improvement of all investigated QOL parameters. Specifically, there was a statistically significant improvement of the burning sensation and oral hygiene measures as early as 6 weeks after PDT treatment extending previous observations showing improvement QOL parameters upon PDT treatment^13,45,46,49^. However, lesion size reduction alone may be a difficult parameter to assess since OLP lesions have irregular forms, and variable clinical phenotypes, i.e. atrophic or/and erosive areas within the same lesion.

Major efforts have been made to develop and validate clinical markers for mucosal lesions in OLP including individual scoring systems^53^ and measurement of lesions with calipers or periodontal probes^46^ and /or scored the lesions using the Thongprasom score^5,13,45–47,49^. To address this issue, we sought to standardize the measurement of OLP lesions by a computer software using clinical photographs on one hand, and by measuring the lesions in two directions (horizontally and vertically) related to fixed intraoral reference points on the other hand. In contrast to previous studies, only one oral lesion per patient was treated to give a single value per lesion size per time point^45,46,53^. This may have been a drawback of the study, when considering the parameter QOL.

The effect of PDT on the oral microbiome has been only minimally investigated. Reports based on studies in mice^54,55^ and humans^56,57^ have shown that photobiomodulation (therapeutical application of low levels of red and/or near-infrarated light) may positively affect the human microbiome^54^. Additionally, studies investigating the effect of antibacterial PDT on oral bacteria have shown that various photosensitizers may exercise various effects on  oral pathogens inducing selective biofilm inactivation^58,59^. Nonetheless, debatable is its efficiency in the deeper parts of the oral biofilm since bacteria embedded in biofilms provide a higher tolerance towards antimicrobials^60,61^. In the present study, bacterial analysis of 18 oral pathogens (*A. actinomycetemcomitans, A. viscosus, T. forsythia, C. rectus/showae, T. denticola, E. corrodens, P. intermedia, P. micra, P. gingivalis, F. nucleatum, A. odontolyticus, Capnocytophaga sp., C. concisus, E. nodatum, S. constellatus group, C. gracilis, S. mitis group, P. nigrescens, S. gordonii group, V. parvula)* was performed from saliva samples before and (Table 3) two weeks after PDT. Despite a reduction of the majority of bacteria, none was statistically significantly reduced two weeks after therapy. Thus, in the present study, a significant influence of the oral microbiome by PDT cannot be admitted.

Clear guidelines regarding the recommended photosensitizer or the duration of PDT exposure are currently not available. Some authors used 8 applications in 1 month with 50 µl toluidine blue (1 mg/ml) combined with laser irradiation for 2.5 min (fluence: 1.5 J/cm^2^, power density: 10 mW/cm^2^, 630 nm)^46^. Others used a 5% methylene blue solution combined with diode laser irradiation (power density: 100–130 mW/cm^2^, 660 nm) with 8 applications in 2 month^49^ or with a different light source (LED light, 630 nm, power 7.2–14.4 J/cm^2^) with 4 aplications (days 1, 4, 7 and 14) at different doses with 120 J/cm^2 ^^45,46^. We here used 4 applications of PDT within 2 weeks (days 1, 3, 7, and 14) and chose phenothiazine chloride as photosensitizer combined with a low level laser (fluence: 2.49 J/cm^2^, power density: 200 mW/cm^2^, 660 nm).

PDT was used in the present study based on the so far proven cytotoxic effects, and the induced complex inflammatory and immune responses^33–38^. PDT treatment led to a significant reduction of the dermal CD4^+^ and CD8^+^ T- cellular infiltrate in OLP lesions which was merely visible on day 28, indicating a diminished inflammatory cell infiltrate. However, whether PDT treatment has a direct impact on lesional T- cells in OLP or whether this observation is a consequence of an overall PDT-mediated reduced inflammatory process, still needs to be further analyzed. Of note, the number of IL-17a-producing lesional T- cells was increased after PDT treatment. This finding is in contrast to a recent observation by our group which suggests that Th1/Th17 cells in lichen planus are pathogenic^52^. However, recent reports demonstrate that PDT treatment by itself can induce Th17 cell accumulation^62^. As peripheral blood IL-17a^+^ T -cells were decreased after PDT treatment, this may have been the consequence of a transmigration of anti-inflammatory Th17 cells from peripheral blood into the mucosal tissue upon resolution of PDT-induced mucosal inflammation. Further studies are needed to elucidate the functional phenotype of Th17 cells after PDT treatment in OLP. Along this line, Th17 cells and their associated cytokine IL-17a were elevated in the peripheral blood of OLP patients and were also detected in OLP lesions^63–67^. Under non-inflammatory conditions, Th17 cells are critical for the induction of innate and adaptive host responses at mucosal areas and show a great deal of plasticity with pro- and anti-inflammatory functions^68^. Thus, Th17 transmigration in peripheral blood may point towards a transformation towards an anti-inflammatory phenotype. In contrast to peripheral blood IL-17a^+^ T- cells, the relative number of peripheral γ/δ^+^ T- cells was found to be increased after PDT. As γδ T- cells were also sparsely detected in OLP lesions^69^, their elevated numbers in the periphery subsequent to a reduction of local inflammation suggests a rather pro-inflammatory function.

CCL5 and CXCL10 were described as central chemokines for the recruitment of T- cells into OLP lesions^70,71^. Strikingly, 14 days after PDT treatment of single mucosal lesions, serum levels of CXCL10 were significantly reduced. This is in line with the finding that lesional CD8^+^ T- cells, which had been described as a main producer of CXCL10^71^, were significantly decreased after PDT treatment.

PDT leads to a significant decrease of peripheral blood CD4^+^CD137^+^ and CD8^+^CD137^+^ T- cells in OLP patients indicating a systemic anti-inflammatory effect of PDT with a direct impact on activated CD4^+^ and CD8^+^ T- cell subsets, which are presumably critical in the OLP pathogenesis^72^. CD137 (4-1BB) is a co-stimulatory surface molecule expressed on various immune cells including antigen-activated T- cells^73–75^. The natural counter receptor 4-1BBL is up-regulated on activated antigen-presenting cells particularly on dendritic cells^73^. CD137 ligand-mediated co-stimulation of human T- cells induces CD4^+^ and CD8^+^ T- cell expansion, cytokine release and cytolytic effector mechanisms^74^. The detection of CD137 expression by flow cytometry is described as an effective assay for the detection of alloreactive T cells^76^ and therapies targeting CD137 showed anti-inflammatory effects in autoimmune diseases^77–79^.

Nonetheless, in order to evaluate the real benefit of PDT as compared to other phototherapies and to determine the therapeutic effect of a photosensitizer in OLP, in future studies a control group treated with photobiomodulation at a similar wavelength and power density but without a dye may be taken into consideration.

In conclusion, PDT leads to a significant improvement of clinical and QOL parameters in OLP which are associated with local and systemic anti-inflammatory effects. PDT holds promise as a novel therapeutic option in OLP and may deliver new insights for a better understanding of OLP pathogenesis in general.

## Methods

### Study design and OLP patient cohort

Forty-three patients with OLP were screened for participation in this prospective, case-controlled, clinical intervention trial. Inclusion criteria were: histologically proven OLP with a minimal lesion size of 10 mm, age >18 years. Exclusion criteria were pregnancy, renal insufficiency, HIV-, hepatitis C, and untreated heart disease. Finally, 20 eligible patients (mean age: 62 ± 8.66 years, 17 female, 2 smokers) gave their informed written consent to participate in the study (Table 1;) approved by local Ethical Committee Philipps University Marburg, Germany; identification AZ:57/14, approval on 22.07.2014). The study has also been registered in the publicly accessible primary german register DRKS (Deutsches Register Klinischer Studien, registration date 01.07.2019, number DRKS00017540). The study was conducted according to the Declaration of Helsinki (1964, revision 2008) and all study procedures were performed in accordance with its guidelines and regulations.

### Study protocol and evaluated parameters

The most extensive oral lesion was chosen for treatment with PDT which was performed in 4 sessions at days 1, 3, 7, and 14 as shown in Fig. 1. In detail, HELBO^®^ Blue Photosensitizer* was applied and left for 3 min on a previously dried mucosal OLP lesion. After thorough rinsing with sterile saline solution, the lesion was irradiated with a spot probe (HELBO^®^ 2D Spot Probe*) for 30 s/spot (active surface of the spot 19 mm^2^) using a low level laser (HELBO^®^ TheraLite Laser, Bredent® Medical GmbH&Co.KG, Senden, Germany) at an output power of 200 mW/cm^2^ and at an emission of 660 nm.

Lesions were measured and scored at baseline (prior to first PDT; day 1) and 2, 4 and 6 weeks after the last PDT session (day 28, 42, and 56). Lesion size was measured horizontally and vertically at the nearest 0.5 mm with a periodontal probe (PCP UNC-15, Hu-Friedy) and with an image measuring software (ImageJ2) using clinical photographs. Lesions were scored using the Thongprasom score^50^ (0: no lesion, normal mucosa; 1: mild white striae, no erythematous area; 2: white striae with atrophic area <1 cm^2^; 3: white striae with atrophic area ≥1 cm^2^; 4: white striae with erosive area <1 cm^2^; 5: white striae with erosive area ≥1 cm^2^). Treatment evaluation were scored after PDT (day 28, 42, and 56) as proposed by Carozzo and Gandolfo^51^ (complete remission: disappearance of all ulcerative lesions with/without remaining mild striae; partial response: improvement without complete healing of the ulcerative lesions; no response: worsening or absence of any improvement of the lesions).

Additionally, the Autoimmune Bullous Skin Disorder Intensity Scores^80^ (ABSIS) I and II were determined. Moreover, at baseline and 6 weeks after therapy, QOL was assessed for the items pain, burning sensation, impaired food intake and self-performed oral hygiene by means of a *Visual Analogue Scala* (VAS; 0: no symptom, 10: very severe pain/strong impairment).

### Laboratory analyses

For performing flow cytometry, CPDA-treated peripheral blood samples from OLP patients before and 14 days after PDT therapy were stained for 30 min at 4 °C with the following monoclonal antibodies: mouse-anti human CD3-PE-Cy5 (UCHT1), mouse anti-human CD3-APC (UCHT1), mouse anti-human CD4-FITC (RPA-T4), mouse anti-human CD8-FITC (SK1), mouse anti-human CD25-APC (M-A251), mouse anti-human CD127-PE (HIL-7R-M21), mouse anti-human CD137 (4B4-1), mouse anti-human TCR-γδ (B1), mouse IgG_1_-FITC (MOPC-21), mouse IgG_1_-PE (G18-145), mouse IgG_1_-PE-Cy5 (MOPC-21), mouse IgG_1_-APC (G18-145) (all Becton Dickinson, Franklin Lakes, USA), mouse anti-human CCR4-APC (205410), mouse IgG_2_-APC (133303) (all R. & D. Systems, Minneapolis, USA). Red blood cells were lysed by ACK lysis buffer (0.15 M NH_4_Cl, 1 mM KHCO_3_ and 0.1 mM EDTA). Subsequently, cells were washed twice (PBS, 1% BSA, 0.1% NaN_3_), resuspended and analysed by FACS (FACSCalibur, Becton Dickinson, Franklin Lakes, USA). FCS-files were analyzed by FlowJo 7.6 single cell analysis software (FlowJo LLC, Ashland, USA).

The Enzyme-linked immunospot (ELISpot) assays were performed as previously described^81^. IFNγ-, IL-5- and IL-17A- positive spots were developed according to the manufacturers’ instructions (Human IFNγ-ELISpot, Human IL-5-ELISpot, Becton Dickinson, Franklin Lakes, US; Human IL-17A ELISpot Ready-Set-Go, eBioscience, San Diego, US). The ELISpot plates were analysed by an ELISpot plate reader (A.EL.VIS, Hannover, Germany).

For performing the Enzyme-linked immunosorbent assay (ELISA), Citrate phosphate dextrose adenine (CPDA)-treated peripheral blood and saliva samples were centrifuged, and plasma and saliva supernatants were collected and stored at −20 °C. Saliva was collected by spitting method^82^. Saliva was allowed to accumulate and the patients spat every 60 sec over 5 min into a collection tube. Saliva concentrations of the chemokines CCL5, CXCL10 and IL-8 were determined by commercial ELISA (all Biolegend, San Diego, USA).

From the saliva samples collected as previously described, quantitative microbial analysis of 18 oral bacteria (*A. actinomycetemcomitans, A. viscosus, T. forsythia, C. rectus/showae, T. denticola, E. corrodens, P. intermedia, P. micra, P. gingivalis, F. nucleatum, A. odontolyticus, Capnocytophaga sp., C. concisus, E. nodatum, S. constellatus group, C. gracilis, S. mitis group, P. nigrescens, S. gordonii group, V. parvula)* was performed prior and two weeks after therapy by means of real-time PCR using a commercially available test (Firma Greiner Bio-One, Solingen, Germany).

**Figure 6:**
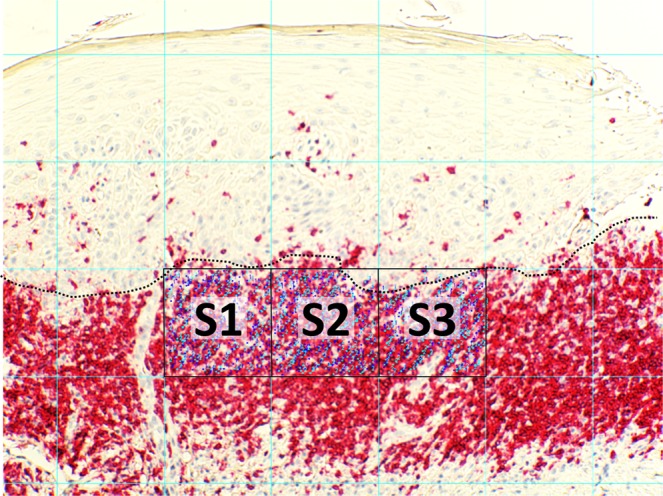
Analysis of T lymphocyte subsets in OLP lesions. The T- cell infiltrate of mucosal sections was quantified by microscopical images (Axiostar, Zeiss, Jena, Germany) in combination with Cell^D (Soft Imaging System, Berlin, Germany) and ImageJ software (freeware). T cells were counted at a 100x magnification. After generating a grid (ImageJ software; area per point: 50.000 pixels^2), all stained T- cells were counted in three squares adjacent to basal membrane zone (ImageJ, cell counter) and their proportion of all infiltrating cells was determined subsequently.

Immunochemistry was conducted on paraffin-embedded skin sections that were processed for immunohistochemistry as recently described^52^. Primary antibodies used were mouse anti-human CD3, CD4, CD8 (all Novocastra, Leica, Wetzlar Germany); rabbit anti-human IL-17A (Novus, Littleton, Co, USA). Secondary antibodies were based on Bond Polymer Refine Detection Kit (Leica, Wetzlar Germany) biotinylated anti-mouse IgG, biotinylated anti-rabbit IgG (Vector Laboratories; Burlingame, CA, USA). Secondary antibodies were subsequently detected by peroxidase- or alkaline phosphatase-labeled ABC-systems (Dako). Visualization was carried out using 3,3’-diaminobenzide (DAB; Dako) as chromogene. The T cell infiltrate of skin biopsies were quantified by microscopical images (Axiostar, Zeiss, Jena, Germany) in combination with Cell^D (Soft Imaging System, Berlin, Germany) and ImageJ software (freeware). CD3^+^, CD4^+^, CD8^+^ and IL-17A^+^ T- cells were counted at 100x magnification. After generating a grid (ImageJ software; area per point: 50.000 pixels^2), all stained/unstained cells were counted in three squares adjacent to basal membrane zone (ImageJ, cell counter; Fig. 6).

### Statistcal Analyses

The study has been registered in the DRKS (Deutsches Register Klinischer Studien), Registration Number DRKS00017540, accepted registration 01.07.2019. (WHO website: http://apps.who.int/trialsearch/).

### Accession code

For statistical analyses the statistical unit was the patient and the main outcome variable was the reduction of the lesion size. Secondary variables were changes in the lesion scores, QOL symptoms, ABSIS I and II scores and changes in the numbers of peripheral blood T- cell subsets (CD8^+^, CD4^+^, CD8^+^CCR4^+^, CD4^+^CCR4^+^, CD8^+^CD137^+^, CD4^+^CD137^+^, IFNγ^+^, IL-5^+^, IL-17a^+^). For all pairwise comparisons of ABSIS scores, lesion size and cell counts between time points generalized least squares (GLS) estimations were used. In the case of the ordinally scaled lesions scores^50,51^ as well as the non-normally distributed QOL scores, non-parametric Friedman test were used to test for a tendency toward higher scores between time points. For the multivariate comparison of QOL scores between day 0 and day 56 the multivariate extension of the Friedman test was applied. P-values were calculated adjusted for multiple comparisons and the level of significance was set at 0.05.

## Supplementary information

Supplementary Figure 1.

## Data Availability

Study protocol data are available https://www.drks.de/ui_data_web/DrksUI.html?locale=de, http://apps.who.int/trialsearch/. Data results are available from arweiler@med.uni-marburg.de, ralucacosgrea@gmail.com.
