# Supplementary material for: Photodynamic therapy in oral lichen planus: A prospective case-controlled pilot study
Source: Sci Rep. 2020 Feb 3;10:1667. doi: 10.1038/s41598-020-58548-9 (PMC6997407; doi:10.1038/s41598-020-58548-9)
Supplement: Supplementary file 1 — Supplementary Figure 1. [file 41598_2020_58548_MOESM1_ESM.docx]

Supplementary Material

**Photodynamic therapy in oral lichen planus:
A prospective case-controlled pilot study.**

**Raluca Cosgarea*, Robert Pollmann, Jusra Sharif, Thomas Schmidt, Ronja Stein, Aura Bodea, Thorsten Auschill, Anton Sculean, Rüdiger Eming, Brandon Greene, Michael Hertl, and Nicole Arweiler**

*** Correspondence:** Raluca Cosgarea: [ralucacosgarea@gmail.com](mailto:ralucacosgarea@gmail.com)

**Supplementary Figures**

**
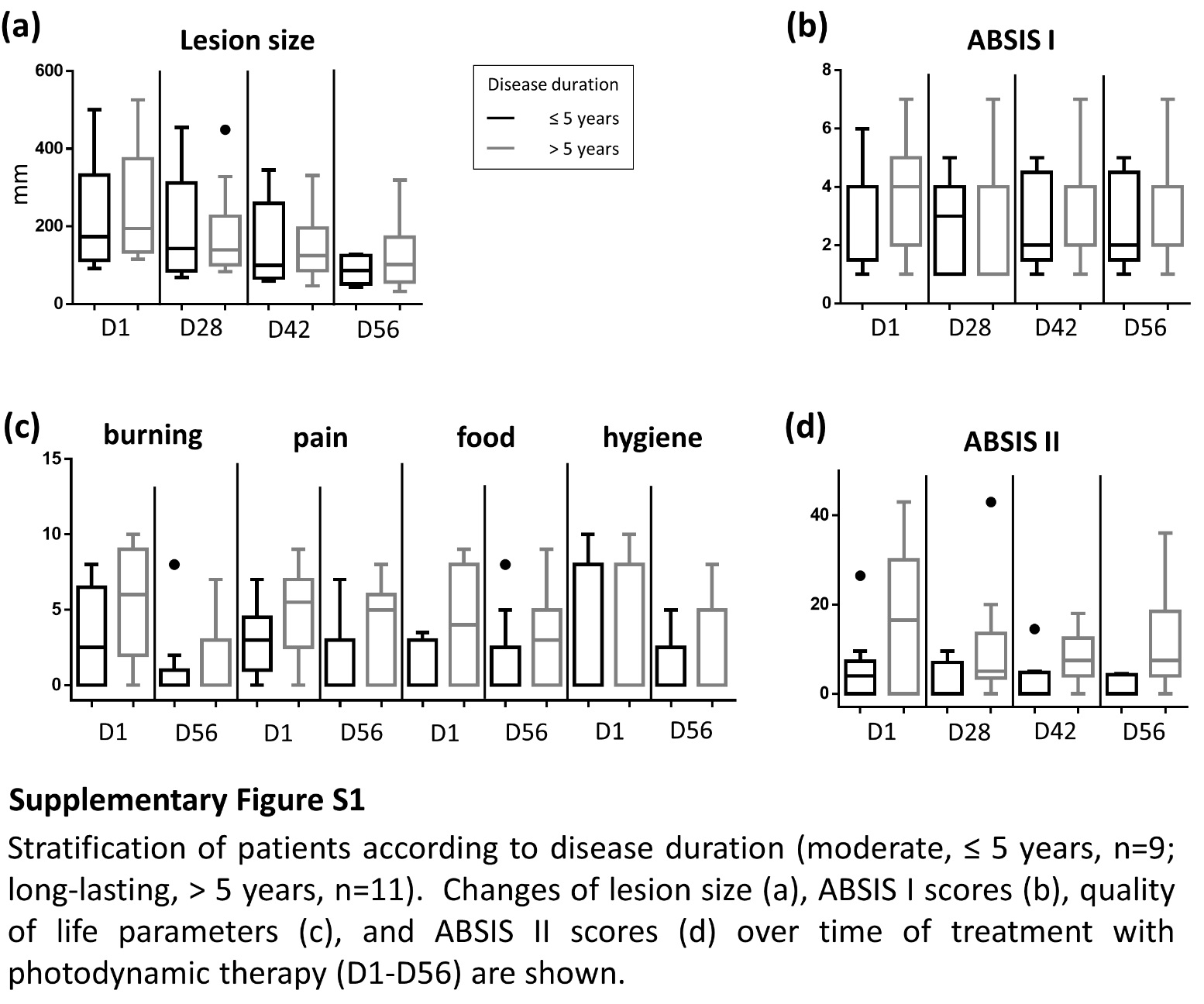
**
